# Local delivery of tranexamic acid for hemorrhage control in trauma: a narrative review

**DOI:** 10.3389/fddev.2026.1771095

**Published:** 2026-02-19

**Authors:** Henry T. Peng, Christian J. Kastrup, Catherine Tenn, Andrew Beckett

**Affiliations:** 1 Defence Research and Development Canada, Toronto Research Centre, Toronto, ON, Canada; 2 Versiti Blood Research Institute, Milwaukee, WI, United States; 3 Department of Surgery, Division of Trauma and Acute Care Surgery, Medical College of Wisconsin, Milwaukee, WI, United States; 4 Departments of Biochemistry, Biomedical Engineering, and Pharmacology and Toxicology, Medical College of Wisconsin, Milwaukee, WI, United States; 5 Michael Smith Laboratories, University of British Columbia, Vancouver, BC, Canada; 6 Defence Research and Development Canada, Suffield Research Centre, Medicine Hat, AB, Canada; 7 St. Michael’s Hospital, University of Toronto, Toronto, ON, Canada; 8 Royal Canadian Medical Services, Ottawa, ON, Canada

**Keywords:** hemostasis, intramuscular injection, local delivery, self-propelling, tranexamic acid, trauma care

## Abstract

**Background:**

Uncontrolled hemorrhage, often accompanied by trauma-induced coagulopathy is a leading cause of preventable death, accounting for 30%–40% of trauma fatalities. Tranexamic acid (TXA), an antifibrinolytic agent, has been extensively studied and proven effective when administered systemically early in severe trauma. However, intravenous (IV) administration poses logistical challenges in prehospital and combat settings, and potential risk of thrombosis. Emerging strategies aim to enhance TXA delivery through localized systems and alternative routes.

**Objective:**

The review explores non-IV routes of delivering TXA for hemorrhage control in trauma, focusing on local, intramuscular (IM), intraosseous (IO), and oral routes, and novel delivery systems.

**Methods:**

A comprehensive synthesis of preclinical and clinical studies was conducted, focusing on the material preparation, characterization, hemostatic efficacy, pharmacokinetics and practical applicability of novel TXA delivery platforms.

**Results:**

Polymeric, inorganic, and composite materials demonstrated enhanced local hemostasis through rapid clot formation and multifunctional properties. Self-propelling systems enabled autonomous penetration into deep wounds, improving clotting time and survival in animal models. IM and IO routes achieved rapid systemic TXA levels comparable to IV as confirmed in both human and animal studies, while oral TXA showed limited utility in acute trauma. Despite promising results, clinical studies on local TXA delivery especially biomaterials-based and self-propelling delivery systems in trauma remain scarce.

**Conclusion:**

TXA demonstrates effectiveness in hemorrhage control through local delivery, either integrated with hemostatic materials or administered via IM and IO routes as practical alternatives to IV infusion in emergency settings. Future research should prioritize formulation optimization, integration of smart features for controlled release, and clinical validation to enable widespread adoption in prehospital and battlefield environments. While topical TXA has shown safety and efficacy in surgical contexts, additional clinical trials are required to confirm its role in traumatic hemorrhage and establish standardized protocols. These innovations offer practical and physiological advantages for managing bleeding, particularly in resource-limited and battlefield environments. Further research is needed to validate safety, scalability, and clinical efficacy across diverse trauma scenarios.

## 1 Introduction

Uncontrolled hemorrhage, particularly non-compressible bleeding from splanchnic and junctional regions, remains the leading cause of preventable death on the battlefield, while tourniquets effectively manage extremity bleeding (Eastridge et al., 2011; Stannard et al., 2013). Globally, hemorrhage accounts for 30%–40% of trauma fatalities among six million victims annually, with half dying before reaching care (Gruen et al., 2012; Kauvar et al., 2006). Trauma-induced coagulopathy (TIC) occurs early, affecting up to one-third of military and one-quarter of civilian patients, and increases mortality three-to five-fold (Davenport and Brohi, 2015; Hess et al., 2008). Notably, 85% of hemorrhagic deaths occur within 6 hours of injury, emphasizing the need for rapid intervention in prehospital settings (Chang et al., 2017; Fox et al., 2017).

Tranexamic acid (TXA), a synthetic lysine analog, inhibits fibrinolysis by blocking plasminogen activation and plasmin-mediated fibrin degradation (Sharma et al., 2012). TXA also blocks the binding of α-2 antiplasmin to plasmin, preventing plasmin activation. Thus, instead of promoting new clot formation, TXA hinders fibrinolysis to reduce bleeding. Beyond antifibrinolysis, TXA may modulate inflammation, protect endothelial integrity, and reduce vascular glycocalyx breakdown (Anderson et al., 2020; Barrett et al., 2020; Lam et al., 2023; Moore et al., 2016; Wu et al., 2017).

It has been reported that TXA reduces mortality in trauma patients if given systemically within the first 3 hours post-injury (Crash-2 collaborators et al., 2011). A meta-analysis of individual patient-level data from 40,138 bleeding patients revealed that immediate treatment with intravenous (IV) TXA improved survival by more than 70%. However, the survival benefit decreased by 10% for every 15-min delay in treatment until 3 hours, after which there was no benefit (Gayet-Ageron et al., 2018). Notably, there was no increase in vascular occlusive events with TXA, with no heterogeneity by site of bleeding, and treatment delay did not modify the effect of TXA on vascular occlusive events.

In recent years, TXA has garnered significant attention for prehospital use in military (Aedo-Martín et al., 2016; Heier et al., 2015; Lipsky et al., 2014; Wright, 2014) and civilian trauma (Almuwallad et al., 2021; Ausset et al., 2015; Stansfield et al., 2020). Despite certain limitations (Da Luz et al., 2012), the overall available data, including the CRASH-2 (Clinical Randomisation of an Antifibrinolytic in Significant Haemorrhage 2) (Crash et al., 2010) and the World Maternal Antifibrinolytic Trial (Shakur et al., 2017), corroborate the efficacy and safety of TXA. These studies suggest the early use of TXA in various clinical settings to reduce blood loss, irrespective of the type of surgery and bleeding volume, offering a survival advantage to many patients (Roberts, 2016).

Although the scientific and historical underpinnings of the CRASH-2 trial have been analyzed alongside non-randomized controlled trials (non-RCTs), randomized controlled trials (RCTs) have been proposed to address the well-described “knowledge gaps” of the CRASH-2 trial (Binz et al., 2015). Ker conducted a systematic review and meta-analysis of the effects of TXA on surgical, traumatic and obstetric bleeding (Ker, 2018). Additionally, a systematic review of RCTs evaluated the effectiveness of TXA in trauma patients (Meza Monge et al., 2024).

Overall, the available data support the efficacy and safety of TXA and suggest its implementation in the prehospital setting for a survival advantage to many trauma patients (El-Menyar et al., 2018). However, the potential risk of venous thromboembolisms from IV administration of TXA has been systematically reviewed, indicating the need for further investigation to identify the optimal targeted trauma patients who can gain the maximum survival benefits with minimum risk of thrombotic complications (Nishida et al., 2017).

Huebner et al. recommended a 1 g TXA IV bolus en route to definitive care for high-risk trauma patients, with subsequent doses withheld until hyperfibrinolysis is confirmed by thromboelastography (TEG) (Huebner et al., 2017). Stansfield et al. conducted a literature review and descriptive analysis supporting the use of a loading dose of 1 g of IV TXA, followed by 1 g infusion over 8 h, administered within 3 hours of injury (Stansfield et al., 2020). This approach aligns with the rational use for TXA in trauma as reported by Napolitano et al. (Napolitano et al., 2013).

Furthermore, different TXA dosing strategies via IV administration in cases of traumatic major hemorrhage (1 g bolus only, 1 g bolus plus 1 g infusion over 8 h, and 2 g bolus) have been compared. The clinical outcomes (24-h and 28-day mortality, multiple organ dysfunction syndrome, venous thromboembolism, and 24-h fibrinolysis state) were found to be equivalent across the three different dosing strategies. Thus, a single bolus administration may be preferable to a bolus plus infusion regimen (Gunn et al., 2024).

A systematic review by Picetti et al. summarized various *in vitro* studies of fibrinolytic inhibition by TXA, determining that a minimal concentration of 5 mg/L TXA in the blood is sufficient to exert an antifibrinolytic effect (Picetti et al., 2019). Prehospital TXA use should be selective for acutely injured patients with severe hemorrhagic shock or traumatic brain injury (Boudreau et al., 2019), and its administration should be optimally guided by viscoelastic hemostatic assays (Moore et al., 2016). TXA has been incorporated into the resuscitation protocols for severely bleeding patients in many trauma centres (Schöchl et al., 2014). However, its use should be selective, particularly for adult trauma patients with severe hemorrhagic shock (systolic blood pressure ≤75 mmHg), or with known predictors of fibrinolysis (Napolitano et al., 2013) and ideally guided by viscoelastic tests (Moore et al., 2016).

Systemic administration of TXA following traumatic injury has been associated with multiple organ failure in patients with fibrinolysis shutdown and hyperfibrinolysis phenotypes (Richards et al., 2021). A systematic review and bias-adjusted meta-analysis of randomized controlled trials comparing TXA to a placebo on traumatic injury in the emergency setting demonstrates that TXA use in trauma emergencies leads to a reduction in 1-month mortality, with no significant increase in problematic vascular occlusive events. Administering TXA in the out-of-hospital setting is associated with reduced mortality compared to in-hospital administration (Fouche et al., 2024).

TXA is typically administered intravenously at the generally recommended dose of 1 g bolus followed by 1 g infusion over 8 h. High doses have not been shown to further increase efficacy and could potentially increase side effects (Faraoni and Fenger-Eriksen, 2024). The development of Intramuscular (IM) injection methods, such as using an autoinjector, would allow for immediate treatment on the battlefield where establishing IV access and controlled infusion of drugs is difficult or operationally impossible (Culligan and Tien, 2011; Vu et al., 2018). With increased evidence supporting the use of TXA in patients with traumatic bleeding, including new evidence on the IM route, United Kingdom paramedics are now authorized to give IM TXA in the pre-hospital setting (Omori and Roberts, 2023).

A recent narrative review summarized TXA use across surgical and trauma settings, noting its administration via oral, IV, and topical routes (Ockerman et al., 2021). Topical application was highlighted for reducing bleeding with minimal systemic exposure, though standardized protocols and further research are needed to confirm efficacy and safety in diverse clinical scenarios. Alternative approaches, including oral and localized delivery, have also been explored to achieve sustained antifibrinolytic effects (Jansen et al., 2017).

A Cochrane review of 29 trials (2,612 participants) reported that topical TXA reduced blood loss by 29% and lowered transfusion risk by 45%, though uncertainty remains regarding thromboembolic events, warranting additional high-quality studies (Ker et al., 2013). Systematic reviews and meta-analyses indicate that topical TXA is as effective and safe as IV administration in both surgical and non-surgical contexts (Georgiev et al., 2018; Montroy et al., 2018). For instance, in total joint replacement, topical and IV TXA demonstrated comparable efficacy without increasing deep vein thrombosis risk (Georgiev et al., 2018). Similarly, meta-analyses show topical TXA improves surgical field visibility and reduces blood loss in endoscopic sinus surgery (Kang and Hwang, 2020). These findings suggest benefits of topical TXA for bleeding patients. This review examines current literature for local TXA delivery strategies for hemorrhage control in trauma.

## 2 Methods

### 2.1 Search strategy

We conducted a comprehensive search using PubMed, Web of Science, and the first 200 hits from Google Scholar to identify studies on the local application of TXA for hemorrhage control in trauma. The search terms included “tranexamic acid,” “aminomethyl cyclohexanecarboxylic acid,” “amino methyl carboxylic acid,” “amino methyl cyclohexane acid,” “cyklokapron,” along with various application methods such as “topical,” “local,” “intramuscular,” “intraosseous,” “subcutaneous,” “transdermal,” and “oral,” combined with terms like “hemorrhag*” or “haemorrhage*” and “trauma*” or “combat” or “military,” all within titles and abstracts. Medical Subject Headings (MeSH) used in the search included “pre-hospital,” “coagulopathy,” “bleeding,” and “trauma”. The search was not limited by date (up to April 2025), language, or publication status. Additional studies were identified through reference lists and citation tracking.

### 2.2 Study eligibility criteria

Original studies were eligible if they evaluated local or non-systemic TXA administration for traumatic hemorrhage. We excluded studies related to burns, musculoskeletal injuries, pulmonary injuries, and brain injuries (including spinal injuries). Additionally, studies addressing surgical bleeding including orthopedic surgery (Lin and Woolf, 2016), hip arthroplasty (Wang et al., 2015), spine surgery (Izima et al., 2023) or using TXA for other conditions, such as melasma (Liang et al., 2024), were not included. Review articles were excluded unless they specifically focused on or were directly related to hemorrhagic trauma. Commentaries, editorials, and protocols were also excluded. Finally, literature lacking *in vitro* and *in vivo* analyses of hemostatic properties or outcomes was not considered.

### 2.3 Data abstraction

Titles and abstracts were screened for relevance, followed by full-text review. Data were extracted using a standardized form capturing:

- Study characteristics (title, authors, year, design: *in vitro*, *in vivo*, human).
- Intervention details (route, dosage, methodology, sample size).
- Outcomes (hemostatic performance, pharmacokinetics for IM, IO, oral TXA).

Screening and data extraction were conducted online using Covidence (Veritas Health Innovation Ltd., Melbourne, VIC, Australia). Studies were categorized by delivery system and route of administration. Comparative analysis was conducted within and across studies to identify performance trends, limitations, and research gaps where results were harmonized to standardized endpoints (e.g., bleeding time in min, reduction in blood loss (%), improvement in survival (%), PK parameters).

## 3 Results

**FIGURE 1:**
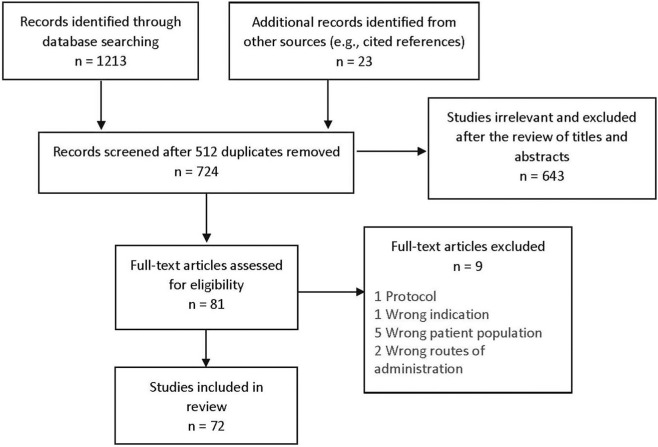
Flow chart of study selection. The screening, full text review and extraction were managed online using Covidence.

A total of 724 records were identified through database searches and other sources (Figure 1). After screening titles and abstracts, 81 studies met the initial inclusion criteria. Nine were excluded during full-text review for reasons such as systemic TXA administration or non-traumatic bleeding, resulting in 72 studies included in the final analysis.

**FIGURE 2:**
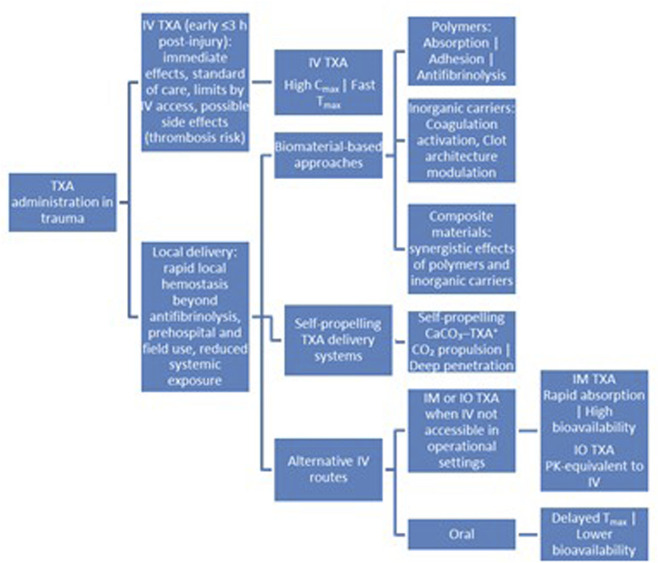
Overview of TXA delivery strategies in trauma care, including conventional IV administration, emerging local biomaterial-based and self-propelling delivery systems, and alternative systemic routes (IM, IO and oral). The schematic highlights the shift from exclusive reliance on IV administration toward local delivery that better meet the logistical and physiological demands of prehospital, battlefield, and resource-limited settings.

Figure 2 illustrates contemporary strategies for TXA administration in trauma, highlighting both established and emerging approaches aimed at improving hemorrhage control. IV TXA remains the standard of care when administered early (within 3 h post-injury), providing immediate antifibrinolytic effects and high systemic exposure. However, its use is limited by the need for IV access and concerns regarding systemic side effects, including potential thrombotic risk.

To address these limitations, three major categories of local TXA application for hemorrhagic trauma have emerged. These approaches aim to provide rapid, site-specific hemostasis beyond antifibrinolysis while reducing systemic exposure, as well as practical alternatives that achieve rapid absorption and high bioavailability, with PK profiles approaching those of intravenous administration—making them particularly suitable for prehospital, battlefield, and resource-limited settings (Figure 2). These categories include: (1) local delivery via biomaterials including polymers, inorganic materials, and their composites (Table 1); (2) self-propelling delivery systems; and (3) IM/IO and oral routes. Biomaterial-based approaches were the most frequently reported (34 studies), followed by self-propelling systems (20 studies) and injectable/oral routes (18 studies).

**TABLE 1:** Biomaterials for local delivery of TXA.

Biomaterial matrix	TXA loading method	Characterization of hemostatic properties	Main findings	References
PVA and chitosan nanofiber mats	TXA (40 mg) added to 100 mL of 7% w/v polymer solution in 2% acetic acid; electrospun at 0.4 and 0.5 mL/h, 27 kV, 12 cm distance	*In vitro* CT and platelet adhesion assessed by inverted tube method and surface interaction of test materials with PRP	TXA significantly reduced CT (by ∼82–65%). Enhanced platelet adhesion and activation observed on TXA-loaded nanofibers	Sasmal and Datta (2019)
PGS/PHEMA porous nanofiber mats	TXA (10% w/w) incorporated via electrospinning with optimized polymer ratios and process parameters	*In vitro* TXA release, cytotoxicity (L929 cells), rat tail-cut bleeding model	TXA-loaded nanofibers showed rapid drug release, high porosity, no cytotoxicity, and significantly reduced bleeding time and volume compared to saline, TXA solution, and blank nanofibers	Varshosaz et al. (2020)
Glutaraldehyde-crosslinked gelatin microparticles	TXA loaded either during gelatin matrix formation (10% w/v) or post-formation via vacuum pressure	*In vitro* CT (thrombus formation), platelet activation (flow cytometry), and *in vivo* liver punch biopsy model	The combination of TXA with gelatin decreased *in vitro* CT while had minimal or no effect on platelet activation. Surface-adsorbed TXA showed superior hemostasis and reduced blood loss compared to Celox® *in vivo*	Aydemir Sezer et al. (2016)
Porous starch powder	TXA–loaded porous starch (TPS) prepared by using starch powder as carrier and TXA as loaded drug component (no detail available)	TEG assays (reaction time R, clotting time K, α angle, maximum amplitude MA): 10–30 mg mixed with citrated human blood; tested in a TEG cup containing 20 μL of CaCl_2_ (0.2 M); rat hepatic trauma model measuring bleeding time and blood loss	TPS reduced R, K, and increased α and MA, indicating faster and stronger clot formation. TPS achieved hemostasis within ∼4 min, reduced blood loss compared to control, and showed no adverse effects *in vivo*	Xi et al. (2018)
Cross-linked microporous starch microspheres (CMSM)	TXA solution mixed with CMSM via vortexing and lyophilization to form TXA-loaded CMSM (TCMSM) (5–100 mg/g loading)	CT, APTT, PT, TT, TEG; rabbit liver and ear artery injury models	TCMSM with 20 mg/g TXA showed shortest CT, APTT, PT, TT, and highest clot strength, outperformed Arista® (positive control), demonstrated antifibrinolytic activity, excellent biocompatibility, and rapid hemostasis in both *in vivo* models	Su et al. (2019)
Iota-CAR/xyloglucan/L-serine powder	TXA (1–2 g) added to polymer solution, freeze-dried, milled, and sieved	*In vitro* WB CT, BCI; mouse tail amputation model	TXA-enhanced powders reduced CT and BCI significantly compared to that of blank powders; strong RBC adhesion observed; *in vivo* bleeding time and blood loss reduced	de Moraes et al. (2022)
Chitosan powder	TXA mixed with chitosan via half-wet grinding	*In vitro* CT determined using rabbit WB (1 mL) mixed with the samples (200 mg) at 37C in a test tube tilted for 30° every 30s. Once the blood was coagulated and the CT was recorded	Chitosan-TXA powder showed pro-coagulant activity; 1:2 mass ratio had shorter CT than Yunnan Baiyao (a Chinese medicine for hemostasis)	Hu et al. (2019)
Alginate/chitosan microparticles	TXA dissolved in water and absorbed into microparticles, followed by lyophilization (2% w/w TXA)	Rabbit liver transection model; bleeding time and blood loss measured	TXA-loaded microparticles significantly reduced bleeding time versus Flashclot and unloaded microparticles; no difference in total blood loss	Li et al. (2012)
HA-DA/CMCS gel powder (heat and Fe^3+^ crosslinked)	Gel powder dispersed in TXA solution (20 mg/20 mL), lyophilized	Rat WB BCI, CT, TEG, rat liver and tail bleeding models	TXA-loaded gel showed lower BCI, CT, stronger clots *in vitro*, and reduced bleeding time and blood loss *in vivo* compared to non-TXA gel	Tang et al. (2024)
Gellan gum microbeads	TXA (30% w/w) dispersed in 1% (w/v) gellan gum solution, followed by a sol–gel transition induced by ionic crosslinking process using AlCl_3_	*In vitro* release and *in vivo* pharmacokinetics in rabbits following oral administration of an aqueous solution of TXA and TXA loaded microbeads in rabbits	Gellan gum concentration influenced release rate; sustained TXA release observed *in vivo*, supporting potential clinical use	Bhattacharya et al. (2013)
Porous starch microparticles	TXA chemically attached to the starch particle via esterification (TXA-SP)	Rabbit WB CT, TEG, rat platelet-deficient plasma APTT, PT; mouse tail amputation and rat liver injury models	TXA-SP showed shortest CT, APTT, PT; reduced TEG R, increased α; shortest bleeding time and least blood loss in the animal bleeding models	Zhao et al. (2022)
Lactic acid-TXA copolymer powder	TXA copolymerized with lactic acid via melt polycondensation	CT with citrated WB; *in vivo* pork liver incision model	The copolymer prepared at 10% TXA molar feed ratio showed best coagulation, promoted hemostasis and healing	Lin et al. (2020)
HPMC/Carbopol 940 gel	TXA added to gel at 3% (w/w)	IVY bleeding time test in healthy male volunteers	TXA gel significantly reduced bleeding time compared to TXA solution and control	Shiehzadeh et al. (2023)
CMCS/DAQP hydrogel	TXA dissolved in CMCS and mixed with DAQP (30 mg/mL final concentration)	BCI, CT, PT, APTT, platelet/RBC adhesion performed with rabbit blood or plasma; mouse tail amputation, rat liver and femoral artery injury models	TXA reduced BCI, CT, enhanced platelet adhesion without altering PT/APTT, erythrocyte adhesion; TXA-loaded hydrogel demonstrated most reduction in hemostasis time and blood loss *in vivo*	Chen et al. (2025)
PDMAA/CMCS hydrogel	TXA mixed with acrylamide monomer, initiator, crosslinker and CMCS in solution, followed by polymerization and crosslinking	BCI, SEM for RBC adhesion; bleeding time and amount measured in a rat liver injury model	TXA hydrogel reduced BCI, promoted RBC aggregation, and achieved fastest hemostasis time and least blood loss	Wang et al. (2024)
Chitosan/chondroitin sulfate/hyaluronic acid sponge dressings	TXA incorporated at 0.75% w/w into polymeric solution, followed by freeze-drying	Hemoglobin absorbance measured in rat WB to assess clotting rate	TXA synergized with chitosan to accelerate clot formation compared to unloaded dressings	Saporito et al. (2018)
PU sponge	Sponge soaked in 100 mg/mL TXA for 2 h, air-dried for 24 h	*In vitro* BCI and CT; rat tail amputation model: blood loss and hemostasis time measured	TXA-containing PU sponge showed lower BCI, faster clotting, and reduced blood loss and hemostasis time compared to untreated sponge	Darakhshan et al. (2023)
Chitosan sponge with PCL-TXA nanoparticles	TXA (10% w/w) encapsulated in PCL nanoparticles and incorporated into chitosan sponge	*In vitro* CT determined with rabbit WB when there was no significant movement of the blood in a test tube	TXA did not alter CT; chitosan sponge reduced CT compared to control	dos Santos et al. (2022)
Chitosan/pectin macroporous sponge	TXA added to chitosan/pectin solution, freeze-gelation process	*In vitro* BCI, RBC/platelet adhesion, PT/PTT measured with human WB or RBC suspension, platelet rich and poor plasma; mouse liver laceration and rat tail amputation models	TXA sponge enhanced clotting, RBC/platelet aggregation compared to control sponge; outperformed commercial hemostats (Medisponge and Celox®), as measured by blood loss and hemostasis time *in vivo*	Humayun et al. (2025)
Oxidized nanocellulose (ONC) fibers in forms of gel, powder and sponge	TXA grafted to ONC via amide bonds (ONC-TXA)	*In vitro* blood cell aggregation; rat tail amputation and liver injury models	OBNC-TXA sponge showed superior coagulation, reduced hemorrhage, restored hemoglobin rapidly	Bao et al. (2023)
Poly (lactic-*co*-glycol acid) grafted chitosan	TXA grafted to chitosan via amide bond formation	*In vitro* BCI, CT; rat tail amputation and liver injury models	Compared to chitosan, TXA graft reduced BCI and CT significantly; improved *in vivo* hemostasis time and blood loss	Zhu et al. (2025)
PVA-chitosan/superhydrophobic laminated material	TXA (2 mg/mL) loaded into polyalcohol-chitosan sponge, laminated with a hydrophobic layer on each side and dried	*In vitro* BCI; multiple rat trauma models (tail amputation, liver penetration, femoral artery injury, back muscle injury)	TXA-loaded material showed superior hemostasis, reduced blood loss, and faster clotting compared to gauze and other laminated materials without TXA	Yang et al. (2024)
Trilaminate dressing composed of sodium hyaluronate, PVA, к-CAR, ethyl cellulose	TXA added to PVA/κ-CAR solution; freeze/thaw cycles and crosslinking with potassium chloride	*In vitro* WB CT, BCI, platelet adhesion; *in vivo* rabbit cranial bone defect model	TXA-loaded dressing reduced CT, BCI, and hemostatic time significantly versus unmedicated dressing; improved platelet adhesion and clot formation	El Halawany et al. (2024)
Gauze (no details provided)	Gauze soaked in 100 mg/mL TXA solution	Rat liver laceration model; blood volume and mortality assessed	TXA-treated gauze reduced free blood at 48 h and showed lower mortality at 14 days (20% versus 50%) than blank gauze, though not statistically significant	Paydar et al. (2020)
HA/collagen membrane	TXA, HA, and collagen dissolved in water, frozen and lyophilized	*In vitro* CT, PT, PTT, INR with rabbit WB/plasma	TXA-containing membrane showed slower coagulation rate than TXA-free membrane	Alnawwar and Bukhari (2025)
Montmorillonite (MMT) powder	TXA mixed with 2% w/v MMT suspension in pH 4 nitric acid aqueous solution at 0.01 mol of TXA per Gram of MMT, centrifuged, freeze-dried, ground	*In vitro* CT, APTT, PT with rabbit WB/platelet-poor plasma	TXA-MMT shortened CT versus MMT and TXA alone; MMT and TXA-MMT significantly shortened both APTT and PT compared to control	Ma et al. (2022)
Nanoclay microspheres (NM)	TXA (2.5–5 mg/mL) added to 2 wt% silicate powder (Laponite RD), mixed 48 h, freeze-sprayed and freeze-dried	*In vitro* APTT and TEG performed with plasma	TXA-doped NM led to most reduction in APTT and TEG reaction time; highest clot strength observed	Denry et al. (2022)
Zeolite powder	TXA (0.5 g) mixed dry with 0.5 g zeolite	Rat femoral artery puncture bleeding mode	No significant difference in bleeding stop time versus zeolite alone; TXA reduced exothermic reaction	Altop and Zincir (2021)
Gold nanoparticles (AuNPs)	TXA used as reducing and capping agent (TXA-AuNPs)	*In vitro* WB CT, thrombus weight, PT, INR, TEG; *in vivo* coagulation rate in a rat tail amputation model	TXA-AuNPs enhanced coagulation: reduced TEG R and K, increased α and MA; *in vivo* coagulation time 0.87 min TXA-AuNPs versus 6 min for native TXA	Jha et al. (2023)
Polylactic acid (PLA)/ZnO nanofibrous composite	TXA (2 wt%) and ZnO (0.5–2 wt%) added to polylactic acid solution, electrospun	Human WB clotting assay as measured by absorbance at 540 nm	TXA-loaded PLA/ZnO showed lower hemoglobin absorbance and improved clotting versus pure PLA	Molapour Rashedi et al. (2021)
Cellulose-silica aerogel composite	TXA (2% w/w) added to polymer mix, air-dried or freeze-dried	*In vitro* BCI, RBC/platelet adhesion, CT, PTT; *in vivo* rat femoral artery bleeding model	TXA-loaded composite showed best BCI (0.8%), 80% RBC absorption, strong platelet aggregation, fastest coagulation; least bleeding time and blood loss versus commercial controls (Gelita-Cel® and Traumastem®)	Mahmoodzadeh et al. (2021)
Bilayer dressing (Propolis- Polycarbonate urethane and silicone)	TXA suspended in 2.5–7.5 vol% propolis resin layer (T-SP)	Lactate dehydrogenase-based platelet adhesion assay; SEM of clot	7.5% TXA-propolis dressing showed dense fibrin network and enhanced activation within 15 min	Nguyen et al. (2024)
Alginate/nano-hydroxyapatite composite aerogel	TXA (20 mg) added to alginate/nano-hydroxyapatite solution, freeze-dried	*In vitro* WB CT, BCI, plasma RT, platelet adhesion	TXA-aerogel significantly reduced CT, RT, and BCI versus plain aerogel; enhanced fibrin formation and clot strength	El Halawany et al. (2022)
Sodium alginate-Carbopol gel with Fe_3_O_4_ and lavender oil	TXA (10 mg) and Fe_3_O_4_ (30 mg) dispersed in the gel with lavender oil (2 mL)	*In vitro* WB clot formation as measured by hemolysis of free RBC	Gel showed rapid clot formation, no synergistic effect of TXA on clotting	Nqoro et al. (2024)

APTT, activated partial thromboplastin time; BCI, blood clotting index; CAR, carrageenan; CMCS, carboxymethyl chitosan; CT, clotting time; DAQP, dialdehyde quaternized pullulan; HA, hyaluronic acid; HA-DA/CMCS, hyaluronic acid-dopamine/carboxymethyl chitosan; HPMC, hydroxypropyl methylcellulose; INR, international normalized ratio; PCL, polycaprolactone; PDMAA, Poly (*N*,*N*-dimethylacrylamide; PGS, polyglycerol sebacate; PHEMA, polyhydroxyethyl methacrylate; PRP, Platelet-rich plasma; PT, prothrombin time; PU, polyurethane; PVA, polyvinyl alcohol; RBC, red blood cell; RT, recalcification time; SEM, scanning electron microscope; SNAP, S-nitroso-N-acetylpenicillamine; TEG, thromboelastography; TT, thrombin time; TXA, tranexamic acid; WB, whole blood.

### 3.1 Local delivery by biomaterials

This section analyzed 34 studies investigating biomaterials for local delivery of TXA to enhance hemostasis and wound healing. The materials were categorized into polymeric systems, inorganic carriers, and composite materials, each offering unique advantages in clot stabilization, drug release, and multifunctionality.

#### 3.1.1 Local delivery of TXA via polymeric materials

Polymers—including biopolymers (e.g., chitosan, alginate, gelatin, cellulose, hyaluronic acid), and synthetic polymers (e.g., PHEMA, PLGA, PU, PVA)—have been extensively studied for TXA delivery in various formats such as nanofibers, microparticles, hydrogels, sponges, and multilayer dressings. These systems combine TXA’s antifibrinolytic activity with the intrinsic hemostatic and bioadhesive properties of polymers to enhance bleeding control and wound healing.

- Electrospun nanofibers: TXA-loaded PVA/chitosan nanofibers reduced CT to 2.78 min compared to 4.22 min for controls, demonstrating synergistic effects of TXA and chitosan on hemostasis (Sasmal and Datta, 2019). PGS/PHEMA nanofibers loaded with TXA achieved 99% wound closure within 72 h and significantly reduced bleeding time to 0.1 min from 5.5 min for normal saline and ∼4 min for blank nanofibers in rat tail-cut models (Varshosaz et al., 2020).
- Microparticles and powders: Gelatin microparticles with surface-adsorbed TXA achieved faster hemostasis than controls in liver injury models (Aydemir Sezer et al., 2016). TXA-loaded porous starch reduced TEG reaction time and achieved hemostasis in ∼4 min in rat hepatic trauma models, outperforming gauze and matching compound microporous polysaccharide hemostatic powder (CMPHP) (Xi et al., 2018). Similarly, crosslinked microporous starch microspheres containing TXA shortened bleeding time to 1.8 min and 2 min in rabbit ear and liver injury models, respectively compared to 2.3 min and 2.6 min for non-TXA control and 2 min and 2.4 min for commercial hemostat Arista® (Su et al., 2019). Iota-carrageenan/xyloglucan/serine powders were loaded with TXA to achieve simultaneous hemostatic, antibacterial, and antioxidant effects (de Moraes et al., 2022). The TXA-loaded powders resulted in clot formation in 0.42 min versus 0.67 min for controls and reduced bleeding time to 2.2 min from 3.4 for untreated group in mouse tail amputation models, performing similarly to non-TXA control and Celox®. Solid chitosan-TXA powders were successfully synthesized using a half-wet grinding method, with chitosan and TXA as the raw materials (Hu et al., 2019). Notably, the clotting time (CT) of the powder with a 1:2 mass ratio between chitosan and TXA was shorter than that of Yunnan Baiyao, Chinese hemostatic medicine. TXA-loaded chitosan/alginate microparticles achieved shorter bleeding time (1.9 min) compared with commercial products such as a fast-acting styptic powder, Flashclot® (3.1 min), without thermal injury, in a rabbit liver transection model (Li et al., 2012). Multifunctional catechol-modified hyaluronic acid-dopamine/carboxymethyl chitosan porous micropowders were developed for rapid hemostasis and enhanced wound healing (Tang et al., 2024). The excellent blood-clotting ability of the TXA-loaded powder was demonstrated *in vitro* through clotting assays and blood clotting index measurements, as well as *in vivo* in rat liver injury and tail-cutting models. TXA-loaded gellan gum microbeads were prepared via sol–gel transition and ionic crosslinking with AlCl_3_ (Bhattacharya et al., 2013). Sustained TXA release was demonstrated in rabbits following oral administration, though hemostatic effects were not assessed. In addition to the physical method, TXA was chemically attached to polymers. TXA-modified porous starch powder (TXA-SP) was synthesized through esterification of porous starch microparticles with TXA (Zhao et al., 2022). The hemostatic effect of TXA-SP was evaluated through in mouse tail amputation and liver injury models. The results showed significantly better hemostatic performance than the positive control (Quickclean®), reducing blood loss by 71% in the tail amputation model and shortening bleeding time to 1.2 min versus 2.2 min in the liver injury model. TXA was chemically incorporated into polymers through melt polycondensation with lactic acid (LA) (Lin et al., 2020). *In vitro* and liver hemorrhage models demonstrated that these powdery copolymers form a degradable protective membrane on wounds, providing sustained hemostatic effects and promoting healing.
- Hydrogels and sponges: TXA-containing Carbopol/HPMC gels reduced bleeding time more effectively than TXA solution (Shiehzadeh et al., 2023). TXA-loaded CMCS/DAQP hydrogels exhibited injectability, self-healing, and antibacterial properties, reducing bleeding time from 8.0 min to 3.3 min and blood loss by 45% compared to gelatin sponges in a mouse tail amputation model, and reducing bleeding time from 4.1 min to 1.8 min and blood loss by 46% in a rat livery injury model (Chen et al., 2025). In addition to its hemostatic effects as an antifibrinolytic drug, TXA promotes the rapid generation of free radicals and introduces multiple hydrogen bonds into the hydrogel network, resulting in an injectable and robust PDMAA/CMCS hydrogel (Wang et al., 2024). The TXA-induced hydrogel achieved hemostasis within 0.42 min and reduced blood loss to approximately one-tenth of the control—in rat liver bleeding models, while promoting wound closure (83% at 2 weeks). Chitosan acetate sponges incorporating glycosaminoglycans and TXA were prepared by freeze-drying a solution containing all components (Saporito et al., 2018). The combination of TXA and chitosan exhibited a synergistic effect, accelerating clotting and enhancing performance compared to TXA-free controls, while glycosaminoglycans improved adhesion but slightly reduced procoagulant activity. A polyurethane (PU) sponge containing several hemostasis-promoting agents—kaolin, tannic acid, and TXA—was prepared by mixing and curing PU precursors (Darakhshan et al., 2023). The TXA-PU sponge showed burst release followed by sustained release of TXA and superior hemostatic activity *in vitro* and in a rat tail amputation model, with antibacterial properties confirmed by zone-of-inhibition tests. Chitosan sponges containing TXA-encapsulated PCL nanoparticles were developed as a local hemostatic agent, though TXA addition did not significantly enhance performance (dos Santos et al., 2022). A TXA-containing chitosan/pectin-based absorbent macroporous sponge was developed using a cost-effective freeze-gelation method, which leverages the principle of thermally induced phase separation (Humayun et al., 2025). TXA was covalently grafted onto oxidized nanocellulose via amide bond formation, producing degradable nanoscale fibers (ONC-TXA) that were processed into emulsions, gels, powders, and sponges (Bao et al., 2023). The sponge exhibited strong pro-coagulant activity *in vitro* and significantly reduced blood loss in rat models of tail amputation, liver trauma, and muscle hemorrhage, with hemoglobin levels recovering from 128 g/L to 165 g/L within 4 days. A PLGA-chitosan-TXA composite sponge was developed by grafting TXA and poly (lactic-co-glycol acid) (PLGA) onto chitosan to enhance hemostatic performance (Zhu et al., 2025). The sponge exhibited high porosity (∼85%), rapid water absorption, excellent biocompatibility, and significantly shortened CTs (<0.5 min), while reducing bleeding time from 1.6 min to 1.2 min in rat tail injury and from 0.73 min to 0.45 min in mouse liver injury, outperforming chitosan alone in both cases. Mechanistic studies suggest its rapid hemostatic effect involves modulation of platelet and erythrocyte aggregation and fibrin degradation.
- Multilayer dressings and laminates: A TXA-loaded superhydrophilic/superhydrophobic laminate was developed, consisting of a PVA–chitosan sponge core loaded with TXA and chloramphenicol, flanked by hydrophobic layers (Yang et al., 2024). Compared to traditional hemostats, the multilayer laminate improved survival by threefold in rat femoral transection models and minimized secondary wound damage by reducing peeling force to one-eighth that of gauze. Comprising sodium hyaluronate, PVA/kappa-carrageenan, and ethyl cellulose, the TXA-loaded trilaminate dressing reduced coagulation time by 40% and blood clotting index by 60% *in vitro*, and decreased bleeding time by 66% in rabbit bone hemorrhage models (El Halawany et al., 2024).
- Other polymeric systems: A study investigating the use of gauze soaked in TXA solution (500 mg/5 mL) for local hemostasis was conducted in a rat liver injury model (Paydar et al., 2020). While early bleeding control (2–15 min after injury) was similar between TXA- and non-TXA gauze groups, TXA-treated animals showed reduced blood loss by 21% at 48 h, required no additional interventions, and had higher 14-day survival (8/10 vs. 5/10). Histological analysis indicated greater fibrosis and granulation tissue in the TXA group, suggesting improved wound healing. Lyophilized hyaluronic acid/collagen membranes loaded with TXA and/or vitamin K were evaluated for hemostatic and wound healing properties (Alnawwar and Bukhari, 2025). TXA-loaded membranes showed slower clotting and higher cytotoxicity compared to vitamin K-loaded variants, which demonstrated superior wound healing and anti-inflammatory effects.

As summarized in Table 1, besides various polymer matrices, TXA incorporation methods varied across studies. Physical loading techniques included electrospinning, freeze-drying, emulsification, and sol–gel transitions, while chemical conjugation was achieved via esterification or amide bond formation. For instance, TXA was covalently grafted onto porous starch and oxidized nanocellulose or starch-based carriers to enhance stability and hemostatic efficacy (Bao et al., 2023; Zhao et al., 2022). Melt polycondensation with lactic acid produced degradable copolymers forming protective membranes on wound surfaces (Lin et al., 2020). Encapsulation within nanoparticles or microspheres, such as PCL, enabled controlled and sustained drug release (dos Santos et al., 2022). Some systems demonstrated burst release followed by sustained delivery, with release profiles often fitting Fickian diffusion or Korsmeyer–Peppas models (Sasmal and Datta, 2019).

Material characterization of TXA-loaded polymer systems employed advanced techniques such as scanning electron microscopy (SEM), Fourier transform infrared spectroscopy (FTIR), X-ray diffraction (XRD), differential scanning calorimetry (DSC), and nuclear magnetic resonance (NMR) (Bhattacharya et al., 2013; dos Santos et al., 2022; Lin et al., 2020). These analyses confirmed structural integrity, porosity, material compositions and stability, and drug distribution within the polymer matrices. Additionally, key functional properties—including bioadhesion, biocompatibility, drug release kinetics, mechanical strength, and water absorption—have been systematically evaluated for various TXA-polymer delivery platforms (Chen et al., 2025; Saporito et al., 2018; Sasmal and Datta, 2019; Varshosaz et al., 2020).

Hemostatic performance was assessed using multiple *in vitro* and *in vivo* methods, with a primary indicator being the material’s ability to rapidly form a stable clot. Whole blood CT is frequently assessed using the test tube inversion technique, where citrated blood is re-calcified with CaCl_2_ and monitored for coagulation (Hu et al., 2019; Sasmal and Datta, 2019). Another common assay is the Blood Clotting Index (BCI), which measures hemoglobin released from unbound red blood cells—lower BCI values indicate superior clotting efficiency (de Moraes et al., 2022; Tang et al., 2024). Additionally, TEG has been widely employed to quantify clot initiation, strength, and stability for TXA-loaded polymeric systems in various formats (Su et al., 2019; Xi et al., 2018).

Preclinical evaluations have employed diverse animal models, including mice (de Moraes et al., 2022; Zhao et al., 2022), rat (Tang et al., 2024; Varshosaz et al., 2020; Xi et al., 2018), rabbit (Li et al., 2012; Su et al., 2019), with injury types ranging from mild (e.g., tail amputation (de Moraes et al., 2022; Tang et al., 2024; Varshosaz et al., 2020; Zhao et al., 2022)) to severe (e.g., liver trauma) (Li et al., 2012; Paydar et al., 2020; Su et al., 2019; Xi et al., 2018). TXA-loaded delivery systems were compared not only to TXA-free controls but also to commercial hemostats such as CMPHP (Xi et al., 2018), and Quick-acting styptic powder (Flashclot) (Li et al., 2012; Xi et al., 2018), Celox® (Aydemir Sezer et al., 2016; de Moraes et al., 2022) and Quickclean (Zhao et al., 2022). These comparisons consistently demonstrated that polymer-based TXA formulations achieved faster hemostasis and reduced blood loss, often outperforming or matching the efficacy of established products.

Beyond hemostasis, many TXA-loaded polymeric systems demonstrated multifunctional therapeutic benefits. Catechol-modified hyaluronic acid/carboxymethyl chitosan micropowders promoted wound healing and tissue regeneration while maintaining rapid clotting performance (Tang et al., 2024). TXA-based hydrogels showed outstanding mechanical strength, injectability, and adhesion, achieving hemostasis in under 0.5 min and promoting wound closure rates exceeding 80% in infected skin defect models (Wang et al., 2024). PU sponges containing TXA, kaolin, and tannic acid demonstrated antibacterial activity and excellent biocompatibility (Darakhshan et al., 2023). Laminated dressings combining superhydrophilic and superhydrophobic layers improved survival in femoral transection models and minimized secondary tissue damage (Yang et al., 2024).

Despite encouraging preclinical results, further clinical trials are needed to validate safety, efficacy, and scalability. Future research should focus on optimizing polymer compositions, integrating smart responsive elements, and elucidating the mechanisms of TXA-polymer interactions at the biological fluid interface (Guo et al., 2023; Mecwan et al., 2022).

#### 3.1.2 Local delivery of TXA via inorganic materials

Inorganic carriers have emerged as robust platforms for localized delivery of TXA due to their structural stability, high surface area, and ability to modulate coagulation pathways. Only four studies have investigated inorganic systems for TXA delivery, each demonstrating favorable hemostatic and therapeutic outcomes.

TXA-Montmorillonite (MMT) composites were synthesized using intercalation technology, confirmed by FTIR, SEM, and XRD analyses (Ma et al., 2022). These composites exhibited excellent biocompatibility in hemolysis and cytotoxicity assays and significantly reduced clotting parameters, including activated partial thromboplastin time (APTT) and prothrombin time (PT), compared to controls. In a radiation enteritis mouse model, TXA-MMT not only improved hemostasis but also attenuated inflammatory markers (IL-1β, IL-6, TNF-α) and preserved intestinal tissue integrity, highlighting its potential for gastrointestinal bleeding management.

Nanoclay microsphere frameworks (NMFs) incorporating TXA were fabricated by dispersing smectite nanoclay in water, followed by freeze-spray and freeze-drying (Denry et al., 2022). Characterization using SEM, optical microscopy, differential thermal analysis (DTA), and XRD confirmed successful TXA incorporation and controlled release. Both TXA-loaded and unloaded NMFs significantly shortened APTT and enhanced clot stiffness, attributed to the formation of thinner fibrin fibers and denser clot structures, suggesting synergistic effects between nanoclay and TXA.

Zeolite combined with TXA was evaluated in a rat femoral artery bleeding model (Altop and Zincir, 2021). Both zeolite and zeolite + TXA groups achieved significantly shorter bleeding times (0.71 min and 1.1 min) compared to saline controls (1.9 min), although no additional benefit was observed with TXA inclusion. Importantly, the zeolite + TXA mixture produced a less exothermic reaction than zeolite alone, reducing thermal injury risk during application.

Gold nanoparticles capped with TXA (TXA-AuNPs) represent a novel inorganic approach offering rapid hemostatic action and theranostic potential (Jha et al., 2023). TXA-AuNPs (∼46 nm) significantly improved coagulation parameters *in vitro*, including shortened PT, international normalized ratio (INR), reaction time (R), and coagulation time (K), along with increased α-angle and maximum amplitude (MA) in TEG assays. *In vivo*, TXA-AuNPs achieved hemostasis in 0.87 min, compared to 6 min for native TXA. Radiolabeling and toxicity studies confirmed favorable biodistribution, clearance, and safety. The use of biologically derived alkyl amines as reducing and capping agents contributed to enhanced coagulation, positioning TXA-AuNPs as promising candidates for acute bleeding control and real-time monitoring.

#### 3.1.3 Local delivery of TXA via composite materials

Composite biomaterials are engineered to address the multifactorial nature of traumatic hemorrhage by integrating complementary hemostatic mechanisms within a single platform. While single-component materials typically excel in one aspect of hemostasis—such as fluid absorption or platelet activation—composites combine structural, biochemical, and mechanical functionalities. For instance, polymer–inorganic composites can couple rapid plasma absorption with surface-mediated coagulation. When supplemented with tranexamic acid (TXA), these systems not only accelerate clot formation but also inhibit premature fibrinolysis, resulting in faster hemostasis, reduced blood loss, and improved survival in trauma models compared with non-TXA single-component counterparts. This multifunctionality is particularly critical in non-compressible hemorrhage, where reliance on a single hemostatic pathway may be insufficient.

PLA/ZnO nanocomposites were fabricated using electrospinning, producing uniform, bead-free nanofibers (∼90 nm) confirmed by field emission SEM, energy-dispersive X-ray spectroscopy (EDS), and FTIR analyses (Molapour Rashedi et al., 2021). These materials exhibited antibacterial activity against *Escherichia coli* and *Staphylococcus aureus*, biocompatibility with human dermal fibroblasts, and effective blood clotting properties. *In vivo* wound healing studies demonstrated accelerated closure over 7–14 days, indicating strong potential as multifunctional dressings for bleeding and infected wounds.

A cellulose-based nanocomposite dressing system (NDS) crosslinked with citric acid and modified with silica aerogel was synthesized using combined chemical and physical cross-linking (Mahmoodzadeh et al., 2021). The incorporation of silica and freeze-drying produced a highly porous structure (∼70% porosity), enabling rapid blood absorption (up to 60 g/g) and strong tissue adhesion (∼90 kPa). The negatively charged surface and embedded calcium ions facilitated rapid coagulation cascade activation. Compared to commercial hemostatic powders (Gelita-Cel® and Traumastem®), NDS demonstrated superior performance in blood absorption, RBC attachment, clotting time, and partial thromboplastin time (PTT). *In vivo* rat femoral artery injury models showed a 2.13–4.4-fold reduction in bleeding time and blood loss compared with Gelita-Cel® and Traumastem®, along with confirmed biodegradability and absence of inflammation or toxicity after 14 days.

A bilayer wound dressing incorporating TXA suspended in a resinous propolis matrix and S-nitroso-N-acetylpenicillamine embedded in a Carbosil polymeric base was also developed (Nguyen et al., 2024). Platelet adhesion assays revealed enhanced fibrin activation within 15 min, supported by SEM imaging showing dense fibrin networks stabilized by TXA. The dressing achieved >98% antibacterial reduction against *S. aureus* and multidrug-resistant *Acinetobacter baumannii*, positioning it as an emergency dressing for traumatic injuries requiring combined hemostatic and antimicrobial action.

Sodium alginate/nano-hydroxyapatite (SA/nano-HA) composite aerogels loaded with TXA were prepared and optimized using a two-level, three-factor full factorial design (El Halawany et al., 2022). The TXA-loaded composite exhibited high fluid uptake (up to 1867%) and a biphasic TXA release profile (>50% within 30 min, followed by sustained release). *In vitro* studies showed a 69% reduction in whole blood clotting time and an 80% decrease in plasma recalcification time compared to controls. However, fibroblast migration assays indicated delayed wound healing for TXA-loaded aerogels.

Finally, alginate-based topical gels incorporating essential oils (eucalyptus, lavender, rosemary), magnetite (Fe_3_O_4_) nanoparticles, and TXA were formulated (Nqoro et al., 2024). XRD and SEM confirmed Fe_3_O_4_ synthesis, while FTIR indicated no chemical interaction among components. The composite demonstrated physiologically compatible pH (6.8–7.2), good spreadability, and stable viscosity. *In vitro* wound healing assays showed 99% closure by day 3, with significant antibacterial and hemostatic activity, suggesting potential for managing bleeding and infected wounds.

### 3.2 Self-propelling delivery systems

Conventional topical hemostatic agents—such as gauze-based products (e.g., Combat Gauze, HemCon)—are limited by their dependence on manual pressure to remain in place and initiate clotting (Peng, 2020). In high stress scenarios, such as care under fire combat conditions, these agents are often dislodged by blood flow without adequate compression, delaying hemostasis and reducing efficacy. This limitation is critical in non-compressible or anatomically inaccessible wounds (e.g., torso, junctional regions, deep cavities), where manual compression is impractical (Baylis et al., 2016a). Furthermore, many of these products are neither biodegradable nor bioabsorbable, requiring removal and risking rebleeding. Their performance is further compromised in TIC, where clotting mechanisms are impaired.

To overcome these challenges, self-propelling particle systems have been developed for targeted delivery of hemostatic agents (Baylis et al., 2016a; Medina-Sanchez et al., 2018). These systems utilize autonomous propulsion against blood flow, enabling penetration into deep wound sites and direct delivery of antifibrinolytic and procoagulant agents (Baylis et al., 2016a). A widely studied approach incorporates calcium carbonate (CaCO_3_) and protonated tranexamic acid (TXA^+^), which react to generate CO_2_ gas, propelling particles into the wound while simultaneously releasing TXA and calcium ions—critical for coagulation (Li et al., 2020; Liu et al., 2021; Lu et al., 2022; Pan and He, 2017; Qiu et al., 2022).

Preclinical studies demonstrate that thrombin–CaCO_3_–TXA^+^ self-propelling particles significantly reduce blood loss and improve survival in swine models of severe hemorrhage, outperforming Combat Gauze when applied without manual pressure (Baylis et al., 2017; Baylis et al., 2016b; Baylis et al., 2015). TXA^+^-loaded gauze achieved superior fibrinolysis inhibition compared to non-protonated TXA formulations (Baylis et al., 2019). Systemic absorption of TXA from these dressings reached therapeutic plasma concentrations (∼10 μg/mL) within 30 min and sustained levels for up to 180 min—sufficient to inhibit fibrinolysis and mitigate hyperfibrinolysis-associated mortality (Ali-Mohamad et al., 2023; Moore et al., 2014; Picetti et al., 2019). Additionally, calcium ions delivered by the dressing is an important cofactor for coagulation enzymes and may help counteract trauma-induced hypocalcemia, a common contributor to coagulopathy (Vasudeva et al., 2021).

Recent innovations include percutaneously deliverable self-propelling powders, which improved survival in swine models of non-compressible intra-abdominal hemorrhage (Cau et al., 2022a; Cau et al., 2022b). These findings underscore the potential of self-propelling systems in managing internal bleeding where traditional methods are ineffective.

The CounterFlow system, a notable example, has demonstrated effective hemorrhage control and survival benefits across multiple animal models simulating junctional wounds, surgical bleeding, intra-abdominal hemorrhage, and upper gastrointestinal bleeding, without evidence of thromboembolism or tissue toxicity (Peng et al., 2023). Furthermore, the CounterFlow hemostatic gauze has shown effective hemorrhage control in live tissue simulations, reinforcing its utility for military medics (Ali-Mohamad et al., 2025). Our recent work further showed that in addition to thrombin, large clotting factor fibrinogen can be encapsulated by CaCO_3_ using methods such as water-oil-water encapsulation, precipitation, and gas diffusion, producing micro-size hemostatic particles with excellent self-propulsion capabilities when mixed with TXA^+^ (Peng et al., 2025).

Beyond thrombin–CaCO_3_–TXA^+^ systems, advanced designs include:

- Janus particles (MSS@CaCO_3_T): Featuring flower-like CaCO_3_ crystals grown on microporous starch substrates, enabling direction-selective propulsion and navigation through irregular wound geometries. In rabbit liver and femoral artery hemorrhage models, these particles loaded with thrombin and powered by the internal component CaCO_3_, with the collaborative use of TXA^+^, reduced bleeding times to 0.83 min and 3 min, and biodegraded within 14 days (Li et al., 2020).
- Chestnut-Like Particles (Pro-MAS): Based on macro-acanthospheres coated with CaCO_3_ and doped with TXA^+^, these particles use spiked morphology to interact with RBCs during propulsion, facilitating localized release of procoagulants and platelet activation. *In vivo* studies in rabbit liver and femoral artery hemorrhage models demonstrated hemostasis within 1.5 min–4 min without applying exogenous procoagulants (Liu et al., 2021).
- Dual-driven Janus particles (SEC-Fe@CaT): Developed by *in situ* loading of Fe_3_O_4_ onto sunflower sporopollenin exine capsules (SEC), followed by the growth of flower-shaped CaCO_3_ clusters (Qiu et al., 2022). By combining CO_2_ bubble propulsion (via CaCO_3_–TXA^+^ reaction) with magnetic navigation using Fe_3_O_4_ clusters, these particles achieved hemostasis in 0.5 min (rat femoral artery) and 0.75 min (rabbit liver), outperforming Celox® (1.17 min).
- Multifunctional microcluster colloidosomes: Constructed from CaCO_3_/Fe_2_O_3_ microparticles coated with Arg-Gly-Asp (RGD)-modified polyelectrolyte multilayers and loaded with thrombin and TXA^+^, these systems integrate magnetic navigation with gas propulsion for deep wound penetration (Lu et al., 2022). Upon exposure to the acidic environment generated by TXA^+^, colloidosomes disassemble, releasing thrombin in a burst while CO_2_ bubbles propel the particles forward. RGD modification enhances platelet binding, improving clot localization. In rabbit liver vase-type hemorrhage models, hemostasis was achieved within ∼1 min, with confirmed biodegradability and biocompatibility.
- Gas-jet propelled powders (COL/PS)_4_@CaCO_3_-T-TXA^+^: Incorporating collagen and protamine sulfate coatings with thrombin and TXA^+^, these powders achieved hemostasis in 0.5 min—approximately 20% faster than Celox® in rabbit hepatic hemorrhage models (Zhou et al., 2023).

Liposomes were employed as a model nanocarrier system for targeted delivery of TXA, with their surfaces functionalized using a fibrinogen-mimetic peptide to enhance site-specific binding (Girish et al., 2019). These TXA-loaded liposomes demonstrated resistance to fibrinolytic degradation *in vitro* when exposed to tissue plasminogen activator (tPA)-spiked rat blood. In a rat liver hemorrhage model, the formulation significantly reduced blood loss and improved survival outcomes while maintaining systemic safety. *Postmortem* analysis of excised tissues confirmed the trauma-targeted accumulation and activity of the TXA-loaded lipid nanovesicles, supporting their potential as a precision hemostatic therapy.

Collectively, these self-propelling and multifunctional systems represent a paradigm shift in hemorrhage control. By enabling pressure-independent, targeted delivery of antifibrinolytic and procoagulant agents, they address critical limitations of conventional dressings—particularly in non-compressible, anatomically challenging wounds and coagulopathic conditions. Their demonstrated efficacy in large-animal (swine) models (e.g., the CounterFlow system), combined with biodegradability and safety, positions these technologies as strong candidates for translation into both military and civilian trauma care.

### 3.3 Local delivery by IM and IO injection

In addition to IV administration, TXA is available in IM, IO, and oral formulations (Prudovsky et al., 2022). IM and IO routes have emerged as practical alternatives to IV delivery, particularly in prehospital and combat settings where IV access may be delayed or infeasible (Vu et al., 2018). IM administration, in particular, offers the advantage of being feasible for self- or buddy-administration by laypersons or military personnel using autoinjectors (Culligan and Tien, 2011), and proposals include issuing 1 g TXA autoinjectors to combat troops for immediate use after severe injury (Wright, 2014), as well as 2 g IV or IO flush protocols for field application (Androski et al., 2020).

As shown in Table 2, thirteen studies compared the pharmacokinetics of IV TXA with alternative administration routes—including IM, IO, and oral—across both human and swine models.

**TABLE 2:** Comparison of pharmacokinetics between IV and alternative route TXA in human and swine.

Study design	IV TXA	Non-IV (IM, IO, oral) TXA	Key findings/Clinical implications	References
Healthy male volunteers received 1 g of IV TXA (n = 3), 0.5 g of IM TXA (n = 3), and 0.5 g of oral TXA (n = 10) TXA	C_max_ 60 μg/mL at 15 min, half-life 1.9 h, 80% urinary excretion in 24 h; T	C_max_ 21.2 μg/mL at 0.5 h and 6.0 μg/mL at 2 h after IM and oral administration, half-life 2.0 h for IM and 3.3 h for oral, urinary excretion 76% for IM and 40%–70% for oral in 24 h	TXA is mainly eliminated from the kidney and is absorbed slowly when given orally. Rapid systemic levels achieved with IV and IM routes	Sano et al. (1976)
Healthy man (n = 3) received 0.5 g of IV TXA and 0.5 g of IM TXA on separate occasions	C_max_ 38.90 μg/mL immediately post- injection, biexponential decline	C_max_ 12.36 μg/mL at ∼1 h, half-life ∼2, bioavailability 1.05	IM TXA provides fast and complete systemic bioavailability, comparable to IV administration	Puigdellívol et al. (1985)
Meta-analysis of pharmacokinetic studies in healthy volunteers (n = 10 for IV TXA 0.5–1 g, n = 6 for IM TXA 0.5–1 g, and n = 114 for oral TXA 0.25–2 g)	CL: 7.6 L/h, V_c_: 17.9 L, V_p_: 16.6 L, Diffusional CL: 2.5 L/h	Bioavailability: 1.05 (IM) and 0.46 (oral)	IM TXA achieves near-complete bioavailability and rapid absorption, making it suitable for emergency use Oral TXA has lower bioavailability and slower onset, limiting its role in acute bleeding scenarios	Grassin-Delyle et al. (2019)
Randomised, open-label, crossover trial in 15 healthy volunteers (IV TXA 1 g, IM TXA 1 g (two 5 mL injections, or oral TXA 2 g in solution)	C_max_ of 57.5 mg/L at the end of infusion (∼10 min); duration >10 mg/L: ∼2.9 h; CL: 10.1 L/h; V_c_: 7.7 L; V_p_: 10.8 L	C_max_: 34.4 mg/L (IM) and 12.8 mg/L (oral). Time to reach 10 mg/L: 3.5 min (IM), 66 min (oral with only 11 out of 15 reached). Bioavailability: 1.00 (IM) and 0.47 (oral)	IM TXA achieves rapid and complete systemic exposure, comparable to IV, making it a practical alternative when IV access is delayed. Oral TXA is slower and less complete, limiting its role in acute bleeding scenarios. IM route avoids high IV peaks, potentially reducing dose-dependent adverse effects while maintaining effective levels longer	Grassin-Delyle et al. (2022)
Open-label pharmacokinetic study of 30 bleeding trauma patients (IV TXA 1 g. IM TXA 1 g as two 0.5 g injections ≥90 min after the first injection)	Not detailed in this study	IM absorption constant: 1.94 h^-1^, Bioavailability: 0.77, CL: 7.1 L/h, V_c_: 16.1 L, V_p_: 9.4 L, 5 mg/L reached in ∼4 min, 10 mg/L reached in ∼11 min, duration above 10 mg/L: ∼5.6 h	IM TXA is rapidly absorbed and well tolerated, even in patients with shock, achieves therapeutic levels within min, making it a viable alternative when IV access is delayed, expanding access to TXA in prehospital trauma care and low-resource settings	Grassin-Delyle et al. (2021)
Unhemorrhaged swine (n = 12) randomized to 1 g of IV or IM TXA; swine (n-19) subjected to 35% controlled hemorrhage randomized to 1 g of IV or IM TXA or no treatment, and 15 swine received a 50-mg bolus of tPA at 30 min post-TXA treatment	C_max_ 139.2 μg/mL; T_max_ ∼2 min (124.1 μg/mL and ∼2 min if hemorrhaged)	IM C_max_ 57.3 μg/mL; T_max_ ∼30 min (56.8 μg/mL and 20 min if hemorrhaged); reached equivalent levels by 20 min; sustained >3 h effective in preventing hyperfibrinolysis	IV administration produced higher TXA concentrations during the first 20 min, but IM achieved comparable efficacy thereafter. Hemorrhage did not significantly affect absorption or clearance. IM TXA offers a practical alternative in austere or mass-casualty environments where IV access is challenging	Haberkorn et al. (2024)
Swine (n = 12) subjected to 35% controlled hemorrhage and randomized to receive a 1 g IV TXA infusion over 10 min, or 1 g IM TXA in two 5 mL injections, or 10 mL normal saline IM injection	IV TXA reached C_max_ during the infusion, higher than IM TXA (100 μg/mL versus 63 μg/mL); T_max_ 5 min; AUC 9798 μg·min/mL	IM bioavailability 97%; T_max_ 10 min; AUV 9544 μg·min/mL	IV administration resulted in a higher serum concentration only during the infusion, but both IV and IM routes reversed hyperfibrinolysis completely. no difference in total body exposure to equal doses of TXA between the two routes. IM TXA may prove beneficial in scenarios where difficulty establishing dedicated IV access could otherwise limit or delay its use	Spruce et al. (2020)
Swine (n = 29) subjected to hemorrhagic shock received 15 mg/kg of IV TXA, or 30 mg/kg or 15 mg/kg of IM TXA at two injection sites, or 15 mg/kg of IM TXA at one injection site	IV achieved rapidly therapeutic levels	IM 30 mg/kg matched IV levels; IM 15 mg/kg lower but sufficient; splitting dose across injection sites had no effect	IM TXA at 30 mg/kg recommended when IV access is unavailable; effective for fibrinolysis inhibition; suitable for prehospital/combat care	Lynghaug et al. (2023)
Swine (n = 18) subjected to abdominal/thoracic trauma received 15 mg/kg TXA either IV or IM	C_max_ 48.1 μg/mL; T_max_ 5 min	IM C_max_ 20.9 μg/m; T_max_ 15 min; C_max_ of 36.9 μg/mL and T_max_ 15 min in control without injury; IM TXA concentrations remained above 10 μg/mL for throughout the 85 min protocol as IV TXA	IM administration is effective for inhibition of fibrinolysis despite reduced absorption in shock and viable alternative to IV and IO in shock conditions and prehospital settings	Bakke et al. (2021)
Swine (n = 15) subjected to a simulated blast injury and 40% controlled hemorrhage and uninjured controls (n = 15) randomized to receive 1 g/10 mL TXA via IV infusion, IV push, or IM	IV infusion: T_max_ 10 min (both trauma and control), reached 10 μg/mL in <1 min; IV push: achieved instant therapeutic levels 10 μg/mL, highest C_max_ (up to 727.8 μg/mL in trauma)	IM T_max_: 15 min (control) versus 45 min (trauma) reached >10 μg/mL in 6.4 min (trauma) versus 2.1 min (control), Bioavailability: 0.93 (control) versus 0.52 (trauma); maintained therapeutic levels for 4 h as IV	All methods achieved effective antifibrinolytic levels within 10 min. IV push offers rapid delivery without prolonged IV access. IM slower in trauma but still clinically effective; practical for austere settings. Trauma significantly reduces IM absorption (longer T_max_, lower bioavailability). IM autoinjectors could improve prehospital TXA administration	Wilson et al. (2024)
Swine premedicated with anesthesia received 30 mg/kg of IV TXA (n = 8) and 30 mg/kg of IO TXA (n = 8)	C_max_ of 9.36 μg/mL at 4 min	IO C_max_ of 4.46 μg/mL at 5 min; overall bioequivalent to IV; similar half-life, CL, V_d_ for both routes (≈63–66 min)	IO TXA pharmacokinetically bioequivalent to IV after infusion; viable alternative when IV access is unavailable, especially in emergency or austere settings	Boysen et al. (2017)
Swine subjected to 35% controlled hemorrhage received 1 g TXA diluted in 10 mL saline via IV infusion (10 min), IO bolus, IM bolus (n = 5 per group)	C_max_ 147.8 μg/mL at 5 min, half-life ∼215 min, AUC 10,641.8 μg·min/mL	IO: C_max_ 119.6 μg/mL at 5 min, AUC 8,350.7 μg·min/mL; IM: C_max_ 39.4 μg/mL at 60 min; half-life ∼160 min, AUC 7,811.8 μg·min/mL	IO approximates IV pharmacokinetics; IM slower but achieves therapeutic levels; IO and IM (with autoinjectors) practical alternatives when IV access unavailable	DeSoucy et al. (2019)
Swine (n = 18) subjected to hemorrhagic shock followed by a tPA infusion received an IV or tibial IO infusion of 1 g TXA over 10 min	C_max_ of 160.9 μg/mL at the completion of IV infusion	IO C_max_ of 132.6 μg/mL at completion of the infusion	IO and IV TXA showed similar pharmacokinetics over the 4-h study period and effectively reversed hyperfibrinolysis. IO TXA can be considered in hemorrhagic shock when IV access cannot be established	Lallemand et al. (2018)

AUC, Area under the concentration-time curve; C_max_, Maximum plasma concentration; CL, clearance; tPA, tissue Plasminogen activator; T_an_ T_max_, Time to reach maximum concentration; V_c_, Central compartment volume; V_d_, Volume of distribution; V_p_, Peripheral compartment volume. Effective antifibrinolysis was defined as plasma TXA, concentration ≥10 μg/mL (Grassin-Delyle et al., 2022).

#### 3.3.1 Human studies

Across multiple studies in healthy volunteers, IM TXA consistently demonstrated rapid absorption and near-complete bioavailability. Peak plasma concentrations (C_max_) ranged from 12.36 to 34.4 μg/mL, with therapeutic levels (>10 μg/mL) achieved within minutes (Grassin-Delyle et al., 2019; Grassin-Delyle et al., 2022; Puigdellívol et al., 1985; Sano et al., 1976). In contrast, oral TXA showed slower absorption, lower bioavailability (∼0.47), and delayed time to therapeutic levels, with only a subset of participants reaching effective concentrations within 1 h (Grassin-Delyle et al., 2019; Grassin-Delyle et al., 2022; Sano et al., 1976).

IV TXA achieved the highest and fastest C_max_ values (up to 60 μg/mL), but IM TXA was found to be pharmacokinetically comparable, offering a practical alternative when IV access is delayed or unavailable (Grassin-Delyle et al., 2019; Grassin-Delyle et al., 2022; Puigdellívol et al., 1985; Sano et al., 1976). A meta-analysis confirmed that IM TXA achieves bioavailability close to 1.0, with rapid systemic exposure suitable for emergency use, while oral TXA was deemed suboptimal for trauma scenarios due to its slower onset and reduced systemic exposure (Grassin-Delyle et al., 2019).

Physiologically based pharmacokinetic modeling of TXA in healthy volunteers comparing IV, IM, subcutaneous, and oral routes of administration has reinforced prior findings. Among non-IV routes, IM TXA demonstrated the greatest potential for achieving targeted systemic exposures. Simulations predicted that a 1 g IM dose would result in plasma concentrations exceeding 15 μg/mL within 15 min, with levels maintained above this threshold for approximately 3 h. The estimated systemic exposure (AUC_0_–_6_) ranged from 99 to 105 μg h/mL following a single dose, supporting the feasibility of IM TXA as a rapid and effective alternative to IV administration in acute care settings (Kane et al., 2021).

**FIGURE 3:**
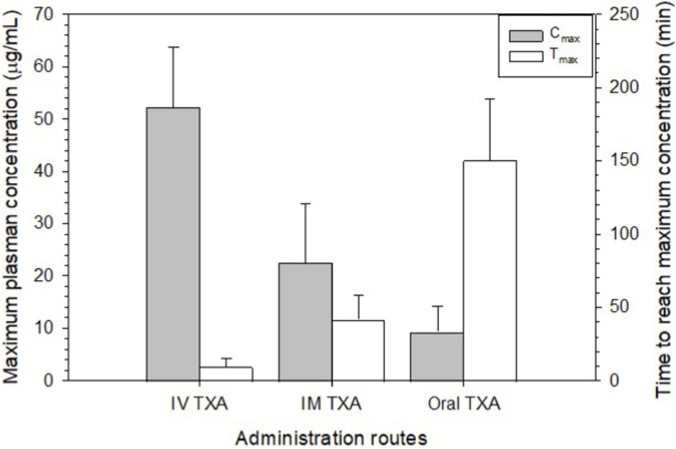
Comparison of key pharmacokinetic parameters among administration routes. Data were pooled from three studies in healthy volunteers (Sano et al., 1976; Puigdellivol et al., 1985; Grassin-Delyle et al., 2022) and calculated as mean ± standard deviation.

Comparison of pharmacokinetic parameters across administration routes based on pooled data from three human studies further demonstrated marked differences in both C_max_ and T_max_ (Figure 3). IV administration resulted in the highest C_max_, with mean peak plasma concentrations exceeding 50 μg/mL, and a rapid T_max_ of approximately 10 min. In contrast, IM administration produced lower peak concentrations, with a C_max_ of approximately 20–25 μg/mL, but achieved this maximum within a relatively short timeframe (≈40–50 min). Oral administration yielded the lowest peak plasma concentrations, with mean C_max_ values below 10 μg/mL, and a substantially delayed T_max_ of approximately 150 min. Across all routes, greater variability in T_max_ was observed for IM and oral administration compared with IV delivery, consistent with inter-individual differences in absorption kinetics. These results highlight a clear route-dependent trade-off between speed of systemic exposure and peak drug concentration.

In trauma patients, IM TXA maintained favorable pharmacokinetics despite physiological stress, reaching therapeutic levels within 4–11 min and sustaining concentrations above 10 μg/mL for over 5 h (Grassin-Delyle et al., 2021). Additionally, a case report highlighted the successful use of IM TXA in a care-under-fire tactical scenario where IV and IO access could not be established, underscoring its practical utility in extreme field conditions (Steele et al., 2022). Collectively, these findings support the viability of IM TXA as an alternative route in resource-limited, prehospital, and combat environments.

#### 3.3.2 Swine studies

Swine models subjected to controlled hemorrhage, hemorrhagic shock, and simulated blast injuries further validated the efficacy of IM and IO TXA. IM TXA achieved therapeutic levels within 6–15 min, with bioavailability ranging from 0.52 in trauma to 0.97 in non-trauma conditions (Bakke et al., 2021; Haberkorn et al., 2024; Lynghaug et al., 2023; Spruce et al., 2020; Wilson et al., 2024). Despite reduced absorption in shock states, IM TXA remained effective in reversing hyperfibrinolysis and maintaining antifibrinolytic levels for several hours (Bakke et al., 2021; Wilson et al., 2024).

IO TXA demonstrated pharmacokinetic equivalence to IV administration, with similar C_max_, T_max_, and AUC (Boysen et al., 2017; DeSoucy et al., 2019; Lallemand et al., 2018). IO delivery was particularly effective in hemorrhagic shock, offering a viable alternative when IV access is compromised.

Across all swine studies, both IM and IO routes successfully reversed hyperfibrinolysis and maintained therapeutic TXA levels, supporting their use in austere, prehospital, and combat settings. Notably, IM TXA at higher doses (30 mg/kg) matched IV efficacy, and autoinjector-based delivery was proposed to enhance field applicability (Lynghaug et al., 2023; Wilson et al., 2024).

In summary, IM and IO routes offer effective, rapid, and practical alternatives to IV administration, particularly in prehospital or austere environments. Their comparable pharmacokinetic profiles and ease of administration make them promising options for early hemorrhage control in trauma care. Both IM and IO achieve therapeutic plasma concentrations within minutes (IV: ∼3–5 min versus IM: ∼5–7 min; IO: ∼7–9 min), whereas oral TXA requires approximately 60 min. The elimination half-life remains consistent across routes at about 2 h (Haberkorn et al., 2024; Spruce et al., 2020; Warner et al., 2025).

## 4 Discussion

This review synthesizes current evidence on TXA delivery strategies for hemorrhage control in trauma, encompassing local administration via biomaterials, self-propelling systems, and alternative injection routes (IM and IO). Collectively, these approaches aim to overcome limitations of IV administration, such as delayed access in prehospital or austere environments, while enhancing site-specific antifibrinolytic action and minimizing systemic risks.

### 4.1 Local delivery via polymeric, inorganic, and composite materials

Polymeric systems—such as sponges, electrospun nanofibers, hydrogels, and multilayered dressings—demonstrate strong hemostatic potential by combining TXA’s antifibrinolytic effect with intrinsic procoagulant properties of polymers through diverse mechanisms. These materials exhibit rapid clotting, high porosity and absorbency, and biocompatibility, with multifunctional features including antibacterial activity and wound healing support. Among them, chitosan-based materials are frequently utilized due to its well-known hemostatic properties and biocompatibility. Inorganic carriers (e.g., montmorillonite, nanoclays, zeolites, gold nanoparticles) offer structural stability and additional benefits such as anti-inflammatory effects and magnetic navigation. Composite systems integrate polymers with ceramics or nanoparticles, achieving synergistic effects—enhanced absorption, mechanical strength, and antimicrobial activity—while enabling sustained TXA release. Preclinical studies confirm that these platforms outperform standard gauze in reducing bleeding time and blood loss, particularly in non-compressible wounds. However, translation to clinical practice requires rigorous evaluation of safety, biodegradability, and scalability.

As summarized in Table 3, biomaterials used for local delivery of tranexamic acid are not passive drug carriers but frequently possess intrinsic hemostatic properties that contribute directly to bleeding control. Common mechanisms include rapid fluid absorption, concentration of clotting factors, platelet adhesion, and formation of a physical barrier that stabilizes nascent clots. For example, polysaccharide-based materials such as porous starch and chitosan promote hemostasis through plasma absorption and electrostatic interaction with erythrocytes and platelets, while collagen- and gelatin-based matrices support platelet activation and fibrin formation. In this context, TXA does not act in isolation but functions synergistically by inhibiting fibrinolysis after clot formation has been mechanically or biologically initiated by the biomaterial. This dual-mechanism approach—structural clot formation combined with biochemical clot preservation—accounts for the superior bleeding control observed in many TXA-loaded biomaterials compared with TXA solution or biomaterials alone.

**TABLE 3:** Roles of biomaterials as intrinsic hemostatic agents in local TXA delivery systems.

Biomaterial class	Examples	Intrinsic hemostatic role	Contribution beyond TXA
Polysaccharides	Cellulose derivatives, chitosan, porous starch, alginate	Physical barrier, contact activation, plasma absorption, platelet aggregation, ionic crosslinking	Rapid clot initiation, sustained clot stabilization and mechanical sealing
Proteins	Gelatin, collagen	Platelet adhesion and activation	Scaffold for fibrin formation
Synthetic polymers	PU, laminates	Mechanical tamponade, wound conformity	Pressure-independent hemostasis
Inorganic materials	Montmorillonite, kaolin, silica	Surface-induced coagulation cascade	Enhanced clot formation and architecture

### 4.2 Self-propelling delivery systems

Self-propelling hemostatic materials were developed to overcome a fundamental limitation of conventional topical agents: their inability to remain at the bleeding site under high-flow or non-compressible conditions. In non-compressible hemorrhage, particularly torso or internal bleeding, blood flow can rapidly displace powders or gauze before effective clot formation occurs. Propelling systems, such as calcium carbonate–based particles loaded with TXA^+^, generate localized gas formation or directional force upon contact with blood, enabling particles to penetrate upstream against blood flow and deliver hemostatic and antifibrinolytic agents directly to the bleeding source (Baylis et al., 2016a). Coupled with intrinsic hemostatic components (e.g., thrombin, fibrinogen, or calcium ions), these systems achieve rapid mechanical clot formation while TXA preserves clot integrity by suppressing fibrinolysis.

Large-animal trauma studies demonstrate that self-propelling systems uniquely combine rapid local hemostasis with delayed but clinically meaningful systemic antifibrinolysis, bridging the gap between topical materials and systemic TXA administration (Ali-Mohamad et al., 2023). These technologies represent a paradigm shift for topical hemostasis in anatomically challenging injuries. Systems incorporating CaCO_3_ and protonated TXA generate CO_2_ bubbles for autonomous propulsion, enabling penetration into deep wound cavities without manual pressure. Multifunctional designs—such as Janus particles, chestnut-like macro-acanthospheres, and colloidosomes—combine propulsion with targeted thrombin release, platelet binding, and magnetic guidance. Animal studies consistently report rapid hemostasis and improved survival compared to standard gauze, even under high-flow conditions. These systems also deliver calcium ions, mitigating trauma-induced hypocalcemia, and achieve systemic TXA absorption sufficient to inhibit fibrinolysis.

However, challenges remain. Gas bubble generation often lacks directional control, leading to superficial dispersion and reduced efficiency; acid–base reactions deplete the CaCO_3_ carrier; and the absence of a matrix for blood component aggregation limits clot formation (Li et al., 2020). Additionally, the propulsion force of many synthetic micromotors may be insufficient to overcome high flow rates and the complex composition of blood, restricting their feasibility in the circulatory system (Xu et al., 2020). Magnetic propulsion offers targeted delivery for deep or non-compressible wounds but requires external equipment, limiting applicability for battlefield and prehospital care (Liu et al., 2021). Further challenges include manufacturing complexity, cost, and regulatory approval.

### 4.3 Alternatives to IV administration: IM, IO and oral routes

IV TXA remains the gold standard for rapid antifibrinolytic therapy, achieving peak plasma concentrations within minutes and maintaining therapeutic levels for approximately 3 hours. However, IV access may be delayed in austere environments or during active hemorrhage. IM administration offers a practical alternative, with pharmacokinetic studies in healthy volunteers and trauma patients confirming rapid absorption (therapeutic levels within 3–11 min), near-complete bioavailability (0.77–1.0), and sustained exposure for several hours. IM delivery via autoinjectors offers a practical solution for self- or buddy-administration in combat or prehospital settings, while IO provides a reliable option when IV access is compromised.

Oral TXA exhibits slower absorption (T_max_ 66–120 min) and incomplete bioavailability (0.46-0.47), limiting its role in acute bleeding. Although oral TXA is less efficient pharmacokinetically, it may offer logistical advantages in mass casualty scenarios (Warner et al., 2025) or extreme environments—such as Arctic conditions—where IV, IM or IO access is unreliable (Lowe et al., 2025).

Collectively, the data underscore that route of administration is a major determinant of TXA pharmacokinetics and therapeutic suitability in time-critical hemorrhage control. While IV delivery maximizes speed and exposure, IM and IO administration offers a clinically meaningful compromise that preserves early antifibrinolytic activity with fewer logistical constraints. These findings support continued efforts to optimize IM formulations and delivery systems to expand access to timely TXA treatment, particularly for trauma care in resource-limited and prehospital settings.

### 4.4 Clinical implications and future directions

The convergence of biomaterial-based local delivery, self-propelling systems, and alternative routes to IV infusion addresses critical gaps in hemorrhage management. IM and IO routes provide rapid, reliable systemic exposure, supporting their integration into trauma protocols and military medicine—particularly for austere and prehospital settings where IV access is challenging. Localized TXA delivery via advanced dressings and self-propelling particles offers targeted hemostasis with reduced systemic exposure, potentially minimizing thromboembolic risk. To date, most investigations have focused on formulation development and *in vitro* characterization, complemented by hemostatic evaluation in animal models.

When standardized endpoints (e.g., bleeding time in rabbit liver injury models) are applied, self-propelling systems consistently demonstrate shorter bleeding time (0.5–1.5 min) than biomaterial delivery platforms (1.9–2.6 min). Self-propelling systems uniquely enable rapid local hemostasis, often independent of manual compression, followed by systemic antifibrinolytic exposure. IM and IO TXA primarily suppress fibrinolysis and achieve antifibrinolytic efficacy on the order of minutes, as defined by time to therapeutic plasma concentrations (>10 μg/mL) (Grassin-Delyle et al., 2022), and are therefore complementary rather than competing strategies. IM and IO routes provide speed comparable to IV access in austere settings, whereas local platforms offer direct hemorrhage control when vascular access is delayed or impossible.

IM, IO, and oral TXA have already been explored in human trials and are likely to be prioritized for clinical implementation. However, existing evidence primarily relates to surgical bleeding, where topical TXA has demonstrated both efficacy and safety. Further research is needed to establish standardized protocols and evaluate its effectiveness in traumatic hemorrhage.

Future research should prioritize:

- Clinical trials evaluating efficacy and safety of IM and IO TXA in large and diverse patient populations.
- Optimization of biomaterial formulations for biodegradability, multifunctionality, and cost-effectiveness.
- Development of autoinjectors and prefilled syringes for IM TXA to enable rapid administration by first responders.
- Regulatory pathways for novel self-propelling and composite systems.

In summary, TXA delivery strategies are evolving toward versatile, rapid-acting solutions that combine systemic and local hemostatic mechanisms. Current evidence supports the safe and beneficial use of local TXA for hemorrhage control, but standardized protocols and further research are essential to establish its role in acute trauma care.

## 5 Conclusion

TXA has been extensively studied for local use, both as a standalone agent and in combination with delivery materials. Localized delivery of TXA through biomaterials, and self-propelling systems, as well as alternative injection routes (IM and IO), offers significant advantages over conventional IV administration in trauma care. Polymeric and composite systems provide rapid and targeted hemostasis with reduced systemic exposure, multifunctionality, and wound healing benefits, while inorganic carriers and gold nanoparticles introduce anti-inflammatory and theranostic capabilities. Self-propelling platforms represent a breakthrough for non-compressible hemorrhage, achieving hemostasis within seconds without manual pressure. IM and IO routes deliver systemic antifibrinolytic effects comparable to IV administration, making them practical for prehospital and combat scenarios where IV access is delayed. Despite these advances, challenges remain in optimizing formulations, ensuring biocompatibility, and addressing manufacturing and regulatory barriers. Future research should focus on hybrid systems, smart-responsive materials, and clinical trials to validate safety and efficacy for field deployment, paving the way for next-generation hemostatic interventions in trauma care.

## Glossary

APTT: Activated Partial Thromboplastin Time (min)
AUC: Area Under the plasma Concentration–time Curve
BCI: Blood Clotting Index (%, lower is better)
CAR: Carrageenan
**C**_**max**_: Peak plasma concentration of TXA (µg/mL)
CMCS: Carboxymethyl chitosan
CL: Clearance
CRASH: Clinical Randomisation of an Antifibrinolytic in Significant Haemorrhage
CT: Clotting Time (min)
DAQP: Dialdehyde quaternized pullulan
ED: Emergency Department
HA: Hyaluronic Acid
HA-DA/CMCS: Hyaluronic Acid-Dopamine/Carboxymethyl Chitosan
HPMC: Hydroxypropyl Methylcellulose
IM: Intramuscular
IO: Intraosseous
INR: International Normalized Ratio
IV: Intravenous
LY30: Lysis at 30 min (%)
MA: Maximum Amplitude (mm)
MMT: Montmorillonite
PCL: Polycaprolactone
PDMAA: Poly(*N*,*N*-dimethylacrylamide
PGS: Polyglycerol Sebacate
PHEMA: Polyhydroxyethyl Methacrylate
PK: Pharmacokinetics
PRP: Platelet-rich Plasma
PT: Prothrombin Time (min)
PU: Polyurethane
PVA: Polyvinyl Alcohol
R: Reaction Time (min)
RBC: Red Blood Cell
RCT: Randomized Controlled Trial
RGD: Arg–Gly–Asp peptide
ROTEM: Rotational Thromboelastometry
RT: Recalcification Time (min)
SNAP: S-Nitroso-N-Acetylpenicillamine
SEM: Scanning Electron Microscope
TEG: Thromboelastography
TIC: Trauma-Induced Coagulopathy
**T**_**max**_: Time to Cmax (min)
tPA: Tissue Plasminogen Activator
TT: Thrombin Time (min)
TXA: Tranexamic Acid
**V**_**c**_: Central Compartment Volume
**V**_**d**_: Volume of Distribution
**V**_**p**_: Peripheral Compartment Volume
WB: Whole Blood
